# Usefulness of Texture Analysis in Differentiating Transient from Persistent Part-solid Nodules(PSNs): A Retrospective Study

**DOI:** 10.1371/journal.pone.0085167

**Published:** 2014-01-08

**Authors:** Sang Hwan Lee, Sang Min Lee, Jin Mo Goo, Kwang-Gi Kim, Young Jae Kim, Chang Min Park

**Affiliations:** 1 Department of Radiology, Seoul National University College of Medicine, and Institute of Radiation Medicine, Seoul National University Medical Research Center, Seoul, Korea; 2 Cancer Research Institute, Seoul National University, Seoul, Korea; 3 Department of Biomedical Engineering, Division of Basic & Applied Sciences, National Cancer Center, Gyeonggi-Do, Korea; University of Nebraska Medical Center, United States of America

## Abstract

**Background:**

Early discrimination between transient and persistent par-solid ground-glass nodules (PSNs) at CT is essential for patient management. The objective of our study was to retrospectively investigate the value of texture analysis in differentiating pulmonary transient and persistent PSNs in addition to clinical and CT features.

**Methods:**

This retrospective study was performed with IRB approval and a waiver of the requirement for patients' informed consent. From January 2007 to October 2009, we identified 77 individuals (39 men and 38 women; mean age, 55 years) with 86 PSNs on thin-section chest CT. Thirty-nine PSNs in 31 individuals were transient and 47 PSNs in 46 patients were persistent. The clinical, CT, and texture features of PSNs were evaluated. To investigate the additional value of texture analysis in differentiating transient from persistent PSNs, logistic regression analysis and *C*-statistics were performed.

**Results:**

Between transient and persistent PSNs, there were significant differences in age, gender, smoking history, and eosinophil count among the clinical features. As for thin-section CT features, there were significant differences in lesion size, solid portion size, and lesion multiplicity. In terms of texture features, there were significant differences in mean attenuation, skewness of whole PSN, attenuation ratio of whole PSN to inner solid portion, and 5-, 10-, 25-, 50-percentile CT numbers of whole PSN. Multivariate analysis revealed eosinophilia, lesion size, lesion multiplicity, mean attenuation of whole PSN, skewness of whole PSN, and 5-percentile CT number were significant independent predictors of transient PSNs. (P<0.05) C-statistics revealed that texture analysis incorporating clinical and CT features (AUC, 92.9%) showed significantly higher differentiating performance of transient from persistent PSNs compared with the clinical and CT features alone (AUC, 79.0%). (P =  0.004)

**Conclusion:**

Texture analysis of PSNs in addition to clinical and CT features analysis has the potential to improve the differentiation of transient from persistent PSNs.

## Introduction

Recent advances in CT technology and its increasingly popular utilization in clinical practice have led to a substantial increase in the number of incidentally-detected small pulmonary nodules, one of which is the pulmonary ground-glass nodule (GGN). The malignancy probability of these GGNs has been reported to be higher than that of solid nodules, and part-solid GGNs (PSNs) in particular, have been reported to have a much higher malignancy probability than solid nodules or pure GGNs, ranging from 62.5%–89.6% [1], [2]. In this context, several experts [3], [4], [5] have suggested that PSNs may represent malignancy with a sufficient enough likelihood to warrant aggressive diagnostic workups including surgical resection.

However, recent studies [6], [7] have revealed that approximately 49–70% of incidentally-detected PSNs decreased in size or disappeared within 3 months. So, the challenge is to correctly identify transient PSNs among initially detected PSNs. In one such attempt, Lee et al. [7] reported that transient PSNs could be differentiated with high accuracy using both the clinical features of patients with PSNs and the visual assessment of CT morphologies of detected PSNs, including younger patient age, follow-up detected lesions, blood eosinophilia, multiple lesions, a large solid portion, and an ill-defined lesion border. However, despite of the excellent performance of this method [7], there are still primary concerns regarding the visual assessment of these CT morphological features such as the interpretation reproducibility between observers and even within the same observer.

In this context, computer-aided texture analysis can be a promising method for lesion characterization. Texture analysis is an imaging analysis method that assesses and quantifies lesion characteristics using pixel values and/or their distribution within target lesions, providing a more detailed and reproducible quantitative assessment of lesion characteristics than visual analysis by human observers. Indeed, several texture features such as skewness, kurtosis, entropy, or uniformity have already been reported to be clinically applicable and valuable in the diagnosis of malignancy, treatment monitoring, and prediction of patients' prognosis [8], [9], [10]. In addition, Goh et al. [8] reported that CT texture analysis reflecting tumor heterogeneity is an independent factor associated with time to progression and has the potential to be a predictive imaging biomarker of the response of metastatic renal cancer to targeted therapy. Furthermore, Ng et al. [10] reported that several texture features such as lower entropy, kurtosis, and standard deviation of pixel distribution; higher uniformity and skewness were associated with a poorer 5-year overall survival rate in patients with colorectal cancer.

To our knowledge, however, there have been no studies describing the usefulness of texture analysis in differentiating transient from persistent PSNs. Thus, the purpose of this study was to retrospectively investigate the value of texture analysis in differentiating between pulmonary transient and persistent PSNs in addition to their clinical and CT features.

## Materials and Methods

This retrospective study was approved by the institutional review board of Seoul National University Hospital with waiver of the requirement for patients' informed consent.

### Study population

One radiologist (S.H.L. with 3 years of experience in chest CT) retrospectively searched for individuals with pulmonary PSNs identified on chest CT taken from January 2007 to October 2009, using the electronic medical records and the radiology information system of Seoul National University Hospital. Our study population was selected in the following steps; first, we selected all CT scans, of which the reports included the words “mixed GGO”, “mixed GGN”, “part-solid nodule”, “mixed ground-glass opacity” or “mixed ground-glass nodule”, resulting in 191 patients with 223 PSNs. Then, two radiologists (S.H.L. and C.M.P., with 3 and 13 years of experience in chest CT, respectively) reviewed all CT scans, and selected cases that met the following criteria: (a) PSNs ≥5 mm and ≤3 cm, (b) individuals with at least one thin-section CT study containing PSNs as well as available follow-up CTs so as to determine the temporality of the PSNs. PSNs<5 mm in size were excluded as it was not practical to evaluate these PSNs in terms of visual morphologic features and texture analysis. Finally, 86 PSNs in 77 individuals (mean age, 54.9 years±9.9; range, 26–77 years) constituted our study population. There were 39 men (mean age, 53.4±9.8; range, 26–73 years) and 38 women (mean age, 56.5±10.0; range, 35–77 years).

There were 39 transient PSNs in 31 individuals and 47 persistent PSNs in 46 individuals, among which 30 were pathologically confirmed; 8 adenocarcinomas-in-situ (AIS), and 22 invasive adenocarcinomas. Of the 86 PSNs, 17 were identical to the study population of our previous reports [11], [12].

We defined a PSN as transient when a nodule decreased in size or disappeared at follow-up CT within 3 months, and we designated a PSN as persistent when a nodule remained stable or increased in size over a follow-up period of 3 months or longer. The determination of whether a nodule was transient or persistent was made through the consensus of two observers (S.H.L. and C.M.P.), who assessed the anatomic relationship between the PSN and the vascular and bronchial structure around the nodule, as well as size measurements. We considered a nodule to have decreased in size when the nodule on a subsequent CT had decreased in size by at least 20% compared with the same nodule on a previous CT [7].

### CT examinations

All 77 individuals underwent at least two low-dose thin-section CT examinations. The mean number of CT examinations was 2.76±1.02 (range, 2 to 8), and the mean CT follow-up period was 227.5 days ±287.51 (range, 10–1528 days).

Thin-section low dose CTs used for visual analysis and texture analysis were performed using one of the following three scanners without intravenous contrast material injection; Sensation-16 (Siemens Medical Solutions, Forchheim, Germany; n = 57), Brilliance-64 (Phillips Medical Systems, Netherlands; n = 10), and Lightspeed Ultra (GE Medical Systems, Milwaukee, WI; n = 10) with 120 kVp, 40 mAs, pitch of 0.75–1.0, and collimation of 1–1.25 mm. Images were reconstructed using a medium sharp reconstruction algorithm with a thickness of 1–1.25 mm. CT scans were obtained for all patients in the supine position at full inspiration.

### Analysis of clinical and visual CT features of PSNs

The clinical and laboratory features of patients with PSNs were recorded by one radiologist (S.H.L.) The following clinical data were detailed: (a) patient age and gender, (b) smoking history (non-smoker, current smoker), (d) blood eosinophil count, and (e) C-reactive protein (CRP) level. Laboratory tests performed within 7 days around CT examination were recorded. Among the 77 individuals, the eosinophil count was available in 75; the CRP level was available in 69. Blood eosinophilia was defined as an eosinophil count exceeding 500/mL.

One radiologist (J.M.G. with 21 years of experience in chest CT) analyzed the thin-section CT features of each PSN. The analyzed CT features of each lesion included: (a) lesion location, (b) lesion size, (c) lesion multiplicity (solitary, multiple), (d) solid portion size, and (e) solid proportion.

### Computer-aided texture analysis of PSNs

**Figure 1:**
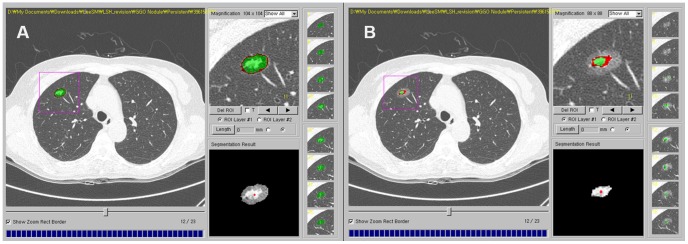
Transverse thin-section CT image showing manual segmentation of a part-solid nodule (PSN). Segmentation of the PSN was manually conducted using an in-house software program and texture features of the nodules were automatically extracted and calculated by the program. One radiologist segmented the outer boundary of the whole PSN (A) and inner solid portion boundary (B).

**Figure 2:**
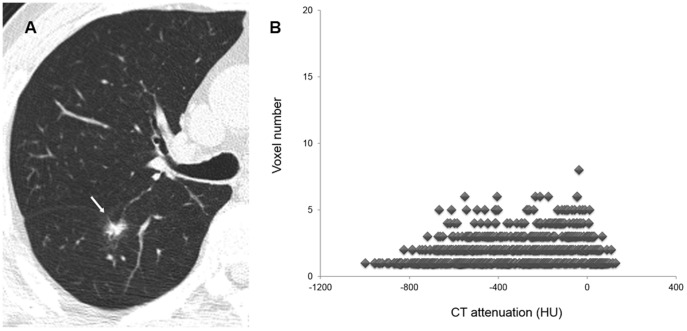
An example of texture analysis of a persistent PSN. (A) Thin-section CT scan shows an 18 mm PSN (arrow) with fissural retraction in the right lower lobe in a 62-year-old male. (B) Texture analysis of the PSN shows high mean attenuation and low negative skewness (−305.5 Hounsfield units and −0.378, respectively). As this PSN was persistent, he underwent lobectomy and was diagnosed as having adenocarcinoma.

**Figure 3:**
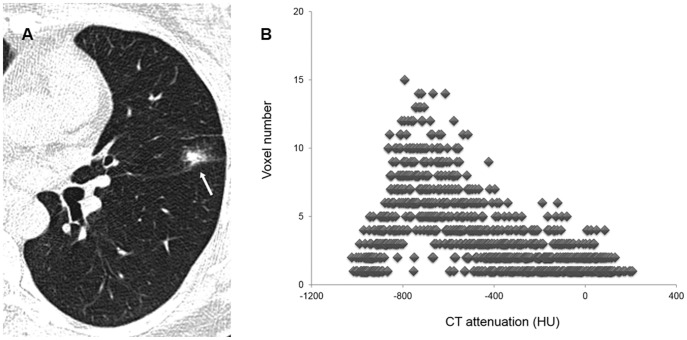
An example of texture analysis of a transient PSN. (A) Thin-section CT scan shows a 17 mm PSN (arrow) with an ill-defined margin in the left upper lobe in a 49-year-old female. (B) Texture analysis of this PSN shows a low mean attenuation and high positive skewness (−570.2 Hounsfield units and 0.856, respectively). The PSN disappeared at follow-up CT after one month.

Thin-section CT images covering PSNs were transferred and stored as DICOM images (16 bit, 512×512 matrix). Nodule segmentation was performed manually using in-house volumetry software by one chest radiologist (S.M.L. with 7 years' experience in chest CT) (Fig 1). Region of interests (ROIs) were drawn around the boundary of each PSN on thin-section CT images until the entire PSN had been covered. After a whole nodule was segmented, various texture features were calculated and extracted automatically. Analyzed texture features were as follows: (a) mean attenuation, (b) standard deviation of attenuation, (c) skewness of attenuation, (d) kurtosis of attenuation, (e) ratio of attenuation of the whole PSN to the inner solid portion, (f) sigmoid fitting slope within the whole PSN, and (g) percentile CT numbers (Fig 2, 3). Skewness is the measure of the asymmetry of the probability distribution of a real-valued random variable in a histogram. Kurtosis is defined as any measure of the “peakedness” of the probability distribution of a real-valued random variable in a histogram, compared to the normal distribution. Sigmoid fitting slope within the entire PSN was made by computing the gray-level profile at each boundary pixel and averaging over all boundary pixels of the contour.

### Statistical Analysis

All statistical analyses were performed using SPSS ver. 18.0 (SPSS Inc., Chicago, IL, USA) and MedCalc ver. 12.0 (MedCalc Software, Mariakerke, Belgium).

Patient age, blood eosinophil count, CRP, thin-section CT features, and texture features of PSNs of the two groups were analyzed using the independent sample *t* test. Statistical differences in patient gender, smoking history, and blood eosinophilia were analyzed using the Pearson **χ^2^** test and the Fisher exact test. In consideration of within-patient correlation, a single PSN was selected randomly prior to nodule evaluation in patients with multiple PSNs.

To identify variables that could be used in differentiating transient from persistent PSNs, logistic regression analysis was performed. Characteristics with a *P*-value <0.05 at univariate analysis were used as independent variables for simple and multiple logistic regression analyses. In multiple logistic regression analysis, an enter method was used, with 5 iterative entries of variables on the basis of test results (*P*-values <0.05). *C*-statistic was also performed to evaluate the performance of the multiple logistic regression model in discriminating transient from persistent PSNs. *C*-statistic equals the area under the receiver operating characteristic curve when the response is binary [13]. This measures how well the models discriminated between transient and persistent nodules. A *C*-statistic of 0.5 indicated no ability to discriminate, while a value of 1.0 indicated perfect discrimination [14]. We also compared the discriminating performance of transient from persistent PSNs between clinical features, thin-section CT features, and the texture features of PSNs.

## Results

### Comparison of clinical and thin-section CT features between transient and persistent PSNs

Table 1 summarizes the clinical features of transient and persistent PSNs. Transient PSNs were more frequently seen in younger patients, in male patients and current smokers than persistent PSNs (P<001, P = 0.001, and P<0.001, respectively). Blood eosinophil count was significantly higher and the presence of eosinophilia was significantly more often found in patients with transient PSNs than patients with persistent PSNs (P<0.001 and P = 0.01). The sensitivity and specificity of blood eosinophilia for transient PSNs were 25.6% and 97.9%, respectively.

**Table 1:** Clinical features in 77 Individuals with Transient and Persistent PSNs.

Characteristics		Transient PSNs (n = 31)	Persistent PSNs (n = 46)	P-value
Age (years)*		50.4±8.6	57.6±9.3	<.001†
Sex (male: female)		22: 9	16: 30	.001‡
Smoking history (non-smoker: current smoker)		19: 12	45: 1	<.001§
WBC (µ/L)*		6331±2511	5691±1300	0.135†
Blood eosinophil (µ/L)*		389.0±289.5	145.3±119.5	<.001†
Blood eosinophilia (≤500 µ/L: >500 µ/L)		22: 7	45: 1	0.01§
CRP*		0.235±0.445	0.242±0.438	0.949†

Note: Except where indicated, data are numbers of individuals.

Data are means ± standard deviations.

Calculated with the independent sample *t* test.

Calculated with the Pearson **χ^2^** test.

Calculated with the Fisher exact test.

PSNs  =  part-solid nodules.

There was a significant difference between transient PSNs (10.9±3.4 mm) and persistent PSNs (14.7±6.3 mm) in lesion size (P<0.001). As for solid portion size, significant differences between transient and persistent PSNs were also observed (P = 0.016). Transient PSNs were significantly more likely to be multiple lesions (P = 0.003) (Table 2).

**Table 2:** CT features in 77 Transient and Persistent PSNs.

Characteristics		Transient PSNs (n = 31)	Persistent PSNs (n = 46)	P-value
Lesion multiplicity (solitary: multiple)		15: 16	37: 9	0.003‡
Lesion location				0.697‡
RUL:RML: RLL		12: 2: 7	22: 3: 12	
LUL: LLL		5: 5	6: 3	
Lesion size (mm)*		10.9±3.4	14.7±6.3	0.001†
Solid portion size (mm)*		4.9±2.6	6.9±4.3	0.016†
Solid portion size (%)*		46.9±21.1	47.9±30.5	0.881†

Note: Except where indicated, data are numbers of nodules.

Data are means ± standard deviations.

Calculated with the independent sample *t* test.

Calculated with the Pearson **χ^2^** test.

PSNs  =  part-solid nodules.

### Comparison of texture features between transient and persistent PSNs

There were significant differences in the texture features of whole PSNs in terms of mean attenuation, skewness of attenuation, ratio of attenuation of whole PSN to inner solid portion, and 5-, 10-, 25-, 50-percentile CT numbers (P = 0.005, 0.009, 0.002, <0.001, <0.001, <0.001, and 0.004, respectively) (Table 3, 4). As for the inner solid portion of PSNs, there were significant differences in 75- and 95-percentile CT numbers (P = 0.027 and 0.047) between the two groups (Table 3, 4).

**Table 3:** Texture Analysis Features in 77 PSNs.

Characteristics	Transient PSNs (n = 31)	Persistent PSNs (n = 46)	P-value
**Whole nodule**			
Mean attenuation	−560.2±135.1	−467.7±138.3	0.005 a
Standard deviation	214.8±54.0	192.4±45.8	0.054 a
Skewness	0.787±0.060	0.4245±0.567	0.009 a
Kurtosis	0.872±2.004	0.300±1.084	0.154 a
Ratio of mean attenuation (whole PSN: inner solid portion)	4.04± 2.20	2.591±1.70	0.002 a
Sigmoid fitting slope	1.16±0.22	1.06±0.35	0.149 a
**Inner solid portion**			
Mean attenuation	−185.8±113.6	−236.7±139.2	0.095 a
Standard deviation	177.2±58.5	154.1±49.6	0.066 a

Data are means ± standard deviations of PSNs' attenuation values.

^a^ Independent sample *t* test

PSNs  =  part-solid nodules.

**Table 4:** The percentile CT numbers in 77 PSNs.

Characteristics		Transient PSNs (n = 31)	Persistent PSNs (n = 46)	P-value
**Whole nodule**				
5 percentile (HU)		−853.3±67.0	−751.0±100.5	<0.001 a
10 percentile (HU)		−809.1±75.1	−700.2±106.8	<0.001 a
25 percentile (HU)		−718.3±101.0	−606.5±125.6	<0.001 a
50 percentile (HU)		−589.2± 144.6	−485.6±154.8	0.004 a
75 percentile (HU)		−426.3±192.0	−343.1±177.4	0.265 a
95 percentile (HU)		−158.8±196.8	−129.4±164.6	0.497 a
**Inner solid portion**				
5 percentile (HU)		−586.0±155.8	−546.2±150.0	0.265 a
10 percentile (HU)		−471.2±146.3	−462.4±142.9	0.792^a^
25 percentile (HU)		−318.2±142.3	−340.7±148.8	0.509^a^
50 percentile (HU)		−160.2±123.5	−223.6±152.5	0.058 a
75 percentile (HU)		−54.7±113.9	−127.7±154.4	0.027 a
95 percentile (HU)		47.0±138.5	−23.1±155.6	0.047 a

Data are means ± standard deviations of PSNs' attenuation values.

^a^ Independent sample *t* test

PSNs  =  part-solid nodules.

### Results of logistic regression analysis in discriminating transient from persistent PSNs

Logistic regression analysis was performed to find independent differentiating variables of transient PSNs from persistent PSNs. Among the clinical and thin-section CT features, age, gender, smoking history, blood eosinophilia, lesion size, solid portion size, and lesion multiplicity were used as input variables for logistic regression analysis. Among the texture features of whole PSNs, mean attenuation, skewness of attenuation, ratio of attenuation of whole PSN to inner solid portion, 5-, 10-, 25-, 50-percentile CT numbers of whole PSN, and 75-, 95-percentile CT numbers of inner solid portion were used for logistic regression analysis.

At multiple logistic regression analysis, clinical and thin-section CT features of eosinophilia, lesion size, and lesion multiplicity proved to be significantly associated with transient PSNs (P = 0.038, 0.022, and 0.020, respectively). Their adjusted odds ratios were 182.905, 0.718, and 37.073, respectively. With respect to texture features of PSNs, mean attenuation, higher positive skewness of attenuation, and 5-percentile CT number of whole PSN proved to be significantly associated with transient PSNs (P = 0.032, 0.043, and 0.007). The adjusted odds ratios were 1.032, 161.826, and 0.952, respectively (Table 5).

**Table 5:** Results of Logistic Regression Analysis for Clinical, Thin-Section CT and Texture analysis of Transient and Persistent PSNs.

Variables	Odds ratio	95% CI	*P*-value
Eosinophilia	182.905	1.349, 24797.948	0.038
Lesion size	0.718	0.542, 0.952	0.022
Lesion multiplicity	37.073	1.760, 780.712	0.020
Mean attenuation†	1.032	1.003, 1.063	0.032
Skewness†	161.826	1.170, 22380.311	0.043
5 percentile CT number†	0.952	0.918, 0.987	0.007

Whole PSN data.

PSNs  =  part-solid nodules

CI  =  confidence interval.

### *C*-Statistics of Predictive Factors

**Figure 4:**
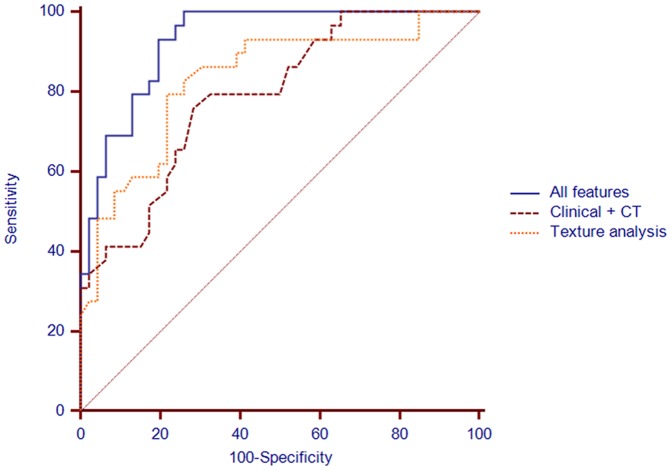
*C-*statistic analysis of multiple logistic regression models in discriminating transient PSNs from persistent PSNs. There were three combinations of independent predictors in the differentiation between transient and persistent PSNs. The highest area under the curve (AUC) was achieved for the combination of clinical, thin-section CT and texture analysis (AUC = 0.929 ? 0.0272). The AUC of clinical and thin-section CT predictors alone (AUC = 0.790 ? 0.0522) was not significantly different from the AUC of computer-aided quantified pixel value predictors alone (AUC = 0.831 ? 0.0503) (P = 0.598). However, the AUC of the combination of clinical, thin-section CT and computer-aided quantified pixel value predictors was significantly higher than that of either the clinical and thin-section CT or the texture analysis alone (P=0.004 and P=0.04). AUCs are shown as means ? standard deviations.

*C*-statistics was performed to evaluate the performance of the multiple logistic regression models in discriminating transient PSNs from persistent PSNs using significant clinical, thin-section CT, and texture features (Fig 4). When independent clinical and CT predictors such as eosinophilia, lesion size, and lesion multiplicity were used as input data, the area under the curve (AUC) was 0.790 (95% confidence interval (CI): 0.681, 0.876). When independent predictors among texture features such as mean attenuation, higher positive skewness of attenuation, and 5-percentile CT number of whole PSNs were used as input data, the AUC was 0.831 (95% CI: 0.727, 0.908). When clinical, thin-section CT and texture features were used as input data, the AUC was measured as 0.929 (95% CI: 0.845, 0.975). There were no significant differences between the AUCs of the logistic regression model incorporating only the clinical and thin-section CT features, and that incorporating only the texture features (0.790 versus. 0.831, *P* = 0.598). However, there were significant differences between the AUCs of the logistic regression model incorporating only the clinical, and thin-section CT features and that incorporating clinical, thin-section CT, and texture features (0.790 versus. 0.929, *P* = 0.004). Similarly, there were significant differences when incorporating only the texture features and that incorporating texture features and clinical and thin-section CT features (0.831 versus. 0.929, *P* =  0.04).

## Discussion

This is the first study to characterize PSNs using texture analysis and we found that texture analysis of whole PSNs has the potential to improve the differentiation of transient from persistent PSNs when used in addition to clinical and CT feature analysis of PSNs. Previously, Lee et al. [7] had shown that PSNs could be differentiated with high accuracy by an expert's visual assessment of thin-section CT features of PSNs. They specifically reported that multiple PSNs, larger solid parts within PSNs, and ill-defined lesion margins were significant discriminators of transient from persistent PSNs. In addition, Oh, et al. [6] found that a spiculated border was another significant differentiating feature of malignant GGNs. However, visual assessment of thin-section CT features has the potential to lean toward interpretation variability between observers and sometimes even within observers [12]. In particular, lesions' margin features such as an ill-defined lesion margin can be significantly dependent on the observer. In this context, texture analysis may have the potential to be a useful method for lesion quantification and characterization.

With respect to the clinical features of PSNs, transient PSNs were more frequently found in patients of younger age, male gender and current smokers, which is consistent with the results of previous studies [6], [7], [15]. Blood eosinophilia is also one of the well-known causes of transient inflammation appearing as transient PSNs [7], [16]. In our study, blood eosinophilia was more frequently found in patients with transient PSNs.

As for CT features of PSNs, smaller lesion size, smaller solid portion size, and lesion multiplicity were more often shown in transient PSNs than in persistent PSNs. The results of clinical and thin-section CT features are consistent with a previous study except lesion size and solid portion size [7].

In regards to significant discriminators of transient from persistent PSNs among clinical and CT features, eosinophilia, lesion size, and lesion multiplicity showed significant differences in multivariate logistic analysis. These results correspond well to a previous study except lesion size [7]. We believe that this difference may result from relatively small study population or different study population. The previous study [7] included only screening individuals.

With respect to the texture features of PSNs, lower mean attenuation, lower 5-percentile CT number and higher positive skewness of attenuation proved to be significant discriminators of transient from persistent PSNs. The lower mean attenuation of transient PSNs can be explained by the fact that transient PSNs frequently show ill-defined margins [7], meaning that the border of the nodule fades out to the adjacent normal lung parenchyma without distinct differentiation. In other words, the periphery of transient PSNs shows little difference compared to that of the normal lung parenchyma. Thus, these pixels can lead to lowered mean attenuation of transient PSNs compared to that of persistent PSNs. These pixels consist of the first few percentiles of attenuation of the whole PSN. In this context, a lower 5-percentile CT number of transient PSNs than in persistent PSNs is to be expected and may be a reasonable result.

In terms of skewness, transient PSNs showed higher positive skewness than persistent PSNs. As skewness is a measure of the asymmetry of the probability distribution of a real-valued random variable in a histogram [17], [18], our results indicate that transient PSNs have the tail on the right side which is longer or fatter than on the left side in a histogram and showed greater asymmetry than persistent PSNs. This can be related to lower mean attenuation, lower standard deviation, and similar attenuation of inner solid portion of transient PSNs compared with persistent PSNs, which eventually lead to more left deviated distribution of attenuation in a histogram.

With logistic regression analysis and *C*-statistics, we found that the differentiating performance of our logistic model using the clinical and thin-section CT features as well as the texture features of PSNs was excellent (AUC, 0.929), and by adding texture analysis of whole PSNs to the classical clinical and thin-section CT features analysis, we were able to significantly increase the differentiating performance between transient and persistent PSNs. We believe that unnecessary procedures such as additional diagnostic tests or invasive procedures may be obviated using this additional analysis method although follow-up CT might not be able to be skipped for definite confirmation of a lesion's persistency.

Our study had several limitations. First, our study was of retrospective design, and was performed on a relatively small number of patients. Second, we retrospectively searched for individuals with pulmonary PSNs identified at low dose thin-section CT using the electronic medical records and radiology information system of our hospital. Thus, there is a possibility that nodules might have been unreported and therefore missed with this search method. Third, the time interval between an initial CT showing PSNs and follow-up CT used for determination of lesions' transiency was not uniform and varied widely. Fourth, the texture features in this study were derived from lesions that were manually segmented by radiologists, which can be significantly influenced by observers' subjective trend or bias. However, manual segmentation may be the gold standard for lesion segmentation particularly in the case of GGNs as their margins are usually indistinct or unclear from the normal lung parenchyma and thus difficult to segment automatically. Nonetheless we believe that a reliable and robust automatic boundary extraction method should be further developed to solve the variability issue.

In conclusion, texture analysis of PSNs has the potential to improve the differentiation of transient from persistent PSNs when used in addition to clinical and CT features analysis.
